# The relationship between personality traits and dysfunctional attitudes in individuals with or without major depressive disorder: a case control study

**DOI:** 10.1186/s12888-023-05392-6

**Published:** 2023-12-04

**Authors:** Jin Liu, Mengqi Zhang, Yumeng Ju, Mi Wang, Yanjun Chen, Jinrong Sun, Xiaowen Lu, Qiangli Dong, Liang Zhang, Ping Wan, Hua Guo, Futao Zhao, Mei Liao, Yan Zhang, Bangshan Liu, Lingjiang Li

**Affiliations:** 1 Department of Psychiatry, National Clinical Research Center for Mental Disorders, and National Center for Mental Disorders, The Second Xiangya Hospital of Central South University, Changsha, 410011 Hunan China; 2 Affiliated WuTaiShan Hospital of Medical College of Yangzhou University, Yangzhou Mental Health Centre, Yangzhou, China; 3 Affiliated Wuhan Mental Health Center, Wuhan, China; 4 Department of Psychiatry, Lanzhou University Second Hospital, Lanzhou, China; 5 Department of Psychiatry, Zhumadian Psychiatric Hospital, Zhumadian, China

**Keywords:** Sixteen personality factor (16PF), Dysfunctional attitudes, Major depressive disorder, Anxiety-related personality traits, Hierarchical regression

## Abstract

**Background:**

Dysfunctional attitudes, which are characterized by distorted self-cognitions, were considered to be linked to personality traits. It was found that certain personality traits may predict dysfunctional attitudes in patients with major depressive disorder (MDD). Nonetheless, the relationship between personality traits and dysfunctional attitudes remains under-researched.

**Aims:**

The aim of this study is to examine the relationship between specific domains of Sixteen Personality Factor (16PF) and dysfunctional attitudes in Chinese participants with or without MDD. In addition, the present study explores the associations between 16PF and eight subtypes of dysfunctional attitudes, based on the proposed eight-factor structure of the Chinese version of the Dysfunctional Attitude Scale-Form A (C-DAS-A).

**Methods:**

One hundred and sixty-eight participants with MDD and 130 healthy participants were included in the study (Trial Registration Number: ChiCTR1800014591). Personality was assessed using the 16PF Questionnaire. Dysfunctional attitudes were measured through the C-DAS-A.

**Results:**

The 16PF dimensions associated with dysfunctional attitudes and the eight subtypes were mainly concentrated in the four anxiety facets including factors C, L, O, and Q4, in both MDD and HC groups. There were significant differences in the 16 PF dimensions that would explain dysfunctional attitudes between the two groups, which were as follows: factors C, G, and O in the MDD group, and factors L and Q4 in the HC group.

**Conclusions:**

Personality traits, especially the anxiety-related personality traits, were distinctly associated with the development of dysfunctional attitudes in people with or without MDD.

## Introduction

 Beck’s cognitive theory of depression indicates that a distorted self-cognition may play a key role in the development and maintenance of depressive illnesses [1]. These distorted cognitions correspond to the dysfunctional attitudes [2], which have been demonstrated as a major vulnerability factor of depression [1]. However, the explanations on the development of dysfucntional attitudes remains an unknown. Studies suggests dysfunctional attitudes may reflect personality traits [3], as well as the previous findings that dysfunctional attitudes in patients with depression can be significantly predicted by specific personality traits [4]. The association between neuroticism traits and dysfunctional attitudes has been well supported [5, 6], giving us a glimpse into this relationship based on the Five-Factor model of personality. However, far less attention is focused on the association between personalities and dysfunctional attitudes from a broader and deeper perspective.

The Sixteen Personality Factor (16PF) Questionnaire developed by Cattell is a comprehensive measurement of normal-range personality for an in-depth evaluation of individuals [7]. The 16PF identified sixteen bipolar conceptualised core traits as well as five broad dimensions of global factors [8]. The five global factors describe primary personality factors at a broader, conceptual level, with discovering the details that may comprehensively explain the uniqueness of an individual’s whole personality [9]. What attracted the attention of the researchers was the global factor anxiety, which consists of four primary factors including emotional stability (C), vigilance (L), apprehension (O), and tension (Q4), indicating the individuals’ high or low anxiety level according to the scores of the four dimensions [10]. According to Cattell et al., the emotional stability (a low score of C factor), suspiciousness (a high score of L factor), self-blaming insecurity (a high score of O factor), and a plethora of nervous energy and drive (a high score of Q4 factor) define the high anxiety [1].

In terms of item content, low C factor scorers show unstable emotion and distorted viewpoints that might produce feelings of poor self-worth and low self-esteem. High L scores adds the psychological discomfort, distrust, insecurity, and alienation. High O factor scorers tend to be seen as worriers, apprehensive, insecure, and self-doubting. High Q4 factor scorers might feel tense and respond with impatience and irritation when experiencing stressful situations [1].

Current research determining the relationship between 16PF and dysfunctional attitudes is limited. However, the characteristics of C factor, L factor, O factor, and Q4 factor clearly correspond to the items of dysfunctional attitudes, such as holding an attitude that “People will probably think less of me if I make a mistake”, “Has a rigid tendency to pursue the approval of others like”, and “My value as a person depends greatly on what others think of me” [2]. Additionally, the content descriptors of the anxiety facets of 16PF have been supported by correlations of the neuroticism facet of the Big Five (NEO PI-R) [1], which might support the potential association between the anxiety facets of 16PF and dysfunctional attitudes.

Therefore, this study aims to examine the relationship between specific domains of 16PF and dysfunctional attitudes in people with MDD. Furthermore, the present study will also investigate this relationship in heathy individuals. Based on the current evidence, it is hypothesized that 16PF domains, especially the anxiety-related personality traits, might be associated with dysfunctional attitudes.

## Methods

### Participants

Data of this study came from a project investigating the biopsychosocial mechanisms of MDD (Project name: *Hypothalamic–pituitary–adrenal axis function and magnetic resonance imaging study of trauma-related depression*. Registration number: ChiCTR1800014591). One hundred and sixty-eight patients with MDD and 130 healthy controls (HC) with intact data of personality and dysfunctional attitudes assessment were included in this analysis. A written informed consent were provided to all the participants before enrollment. Two psychiatrists confirmed the diagnosis of MDD through the Structured Clinical Interview for Diagnostic and Statistical Manual of Mental Disorders, Fourth Edition, Text Revision (SCID-IV-TR). Patients with MDD who had a total score of the 24-item Hamilton rating scale for depression (HAMD_24_) ≥ 20 at baseline and showed moderate or severe depression symptoms were included in the study. Additional inclusion criteria of MDD patients were: (1) age ranges from 18 to 60; (2) without any psychiatric medication within two weeks (six weeks for fluoxetine) prior to enrollment; (3) without diagnosis of any other psychiatric disorders, excluding generalized anxiety disorder; (4) without the history of head injury, neurological disorders, or other internal illnesses.

The 130 participants of the HC group were recruited from local community of Zhumadian, Henan, with a total score of HAMD_24_ ≤ 7. Additional inclusion criteria for the control group including: (1) age between 18 and 60 years; (2) without any psychiatric history; (3) without history of substance abuse or dependence except for tobacco; (4) without without the history of head injury, neurological disorders, or other internal illnesses.

The present study was approved by the Medical Ethics Committees of the Second Xiangya Hospital of Central South University and the Zhumadian Psychiatric Hospital.

### Measures

#### Depression

Depression was assessed by the HAMD_24_ [3]. It has been shown that the HAMD_24_ was commonly used in clinical settings, and the version used in this study was translated by the Shanghai Mental Health Center, with a high level of reliability and validity in the Chinese community [4]. The HAMD_24_ consists of 24 items, including 12 items rated from 0 to 4, 9 items rated from 0 to 2, and 3 items rated from 0 to 3. Higher total scores reflect greater severity of depression, with a range between 0 and 75 and a cutoff score of at least 20 that indicates a moderate depression [4].

#### Anxiety

The present study used the Hamilton Anxiety Rating Scale (HAMA) to measure the intensity of anxiety of the participants. HAMA is a 14-item questionnaire with the total score range between 0 and 56 and the range from 0 to 4 of each item [11]. It has been shown that the Chinese version of the HAMA was a reliable and valid measuring instrument in Chinese samples [12].

#### Dysfunctional attitudes

Dysfunctional attitudes were measured in this study by a Chinese version of the Dysfunctional Attitude Scale–Form A (C-DAS-A). The C-DAS-A is a 40-item self-reporting instrument [2]. Each item consists of a statement about the subject and a 7-point Likert scale indicating the degree of agreement from 1 (fully disagree) to 7 (fully agree). The greater the overall score, the more dysfunctional attitudes there are. The good reliability and validity of the C-DAS-A has been demonstrated in Chinese MDD samples and an eight-factor structure of C-DAS-A was proposed [2, 7]. The eight factors are vulnerability (vulnerable self-confidence such as keeping an attitude), attraction and repulsion (believe that happiness relying on other people’s love), perfectionism (immoderate pursuit of perfection), compulsion (selective or overly generalization), seeking applause (has a rigid tendency to seek the approval of other people), dependence (lack of self-independence), self-determination attitude (casting one’s value to comparison with others), and cognition philosophy (positive attitudes), which were adopted widely in recent research [8, 9]. Chen et al. has reported items classified into each factor [2].

#### Personality

The Sixteen Personality Factor (16PF) Questionnaire has been demonstrated to be an effective assessment of in various settings for measuring the in-depth personality of a person [10]. Consistent evidence of empirical study on the 16PF Questionnaire has indicated its validity in clinical settings [1]. The Chinese version of 16PF Questionnaire has been demonstrated with excellent reliability and validity [13].

### Ethical considerations

The authors assert that all procedures contributing to this work comply with the ethical standards of the relevant national and institutional committees on human experimentation and with the Helsinki Declaration of 1975, as revised in 2008. All procedures and this study involving human subjects were approved by the Ethics Committee of the Second Xiangya Hospital of Central South University [approval number: 2012 (238)] and the Zhumadian Psychiatric Hospital [approval number: 2013 (002)]. Participation in the study was anonymous and totally voluntary. Informed consent was obtained from all the participants in the study. All participants were informed about the aim of the study and gave written consent to participate. Participants were informed of the right to withdraw at any time of the study without consequences. No identifiable information were presented in the study or entered into the database and all data were kept in confidential.

### Statistical analyses

SPSS version 27.0 was used for the analytic procedure. Prior to analyses, missing data were identified. Firstly, independent t-tests were used to assess the differences in the demographic and clinical information between the MDD and HC groups. Secondly, the present study conducted Pearson’s correlation test to examine associations between 16PF and dysfunctional attitudes and subtypes, with a *p* = .10 (two-tailed) for statistical significance. Lastly, the present study performed nine hierarchical regression models. Multicollinearity was tested, and no variables excluded because of collinearity. One hierarchical regression analysis was used to examine the effect of 16PF on dysfunctional attitudes, as well as the other eight hierarchical regression analyses were conducted to identify the specific domains of 16PF that may explain each subtype of dysfunctional attitudes. The three hierarchies of the regression models were as follows: level 1: sex, age, education; level 2: duration of total episodes, duration of current episodes, episode counts, HAMA, HAMD_24_; level 3: 16PF factors that show a significant correlation with dysfunctional attitudes and each subtype. All the correlation and regression analyses were respectively conducted in the MDD group and HC group.

## Results

### Demographic information

A total sample of 298 participants were included in this study. The MDD group included 168 patients, with age ranged from 18 to 58 years (*M* = 35.34, *SD* = 9.53), of which 57.1% were female. The HC group included 130 healthy participants, with age ranged between 18 and 45 years (*M* = 32.08, *SD* = 7.76), of which 56.2% were male. Within the MDD group, the average of onset age of depression was 31.94 years, and the average number of episodes of depression was 2.08. The average scores of HAMD_24_, HAMA, and C-DAS-A total of MDD group were much higher than that of HC group, whereas the average years of education of MDD patients were lower than healthy participants. There were statistically significant differences in all these variables between MDD and HC groups (*p* < .01).

Almost all of the 16 PF factors were significantly different (*p* < .05) between the MDD group and HC group, except for the factors A, I, M, N, Q1, and Q2. As for the anxiety facets of 16 PF, the mean scores of C factor of the MDD group were lower than that of the HC group, whereas the mean scores of L, O, and Q4 factors of the MDD group were higher than the HC group, indicating an overall higher level of anxiety in participants with MDD. Table 1 presents demographic and clinical characteristics of MDD and HC groups.

**Table 1:** Means and standard deviations of major study variables of the MDD group and the HC group

	MDD	HC	t/ χ^2^	*p*
	(Mean ± SD)	(Mean ± SD)		
Age (years)	35.34 ± 9.53	32.08 ± 7.76	3.26	**0.001**
Gender (male/female)	72/96	73/57	4.73	**0.035**
Education (years)	10.29 ± 3.54	11.94 ± 3.32	-4.10	**0.001**
HAMD_24_	31.52 ± 7.40	1.39 ± 1.84	50.84	**0.001**
HAMA	18.22 ± 6.25	1.13 ± 1.90	33.25	**0.001**
Episodes	2.08 ± 1.34	—	—	—
Onset age (years)	31.94 ± 10.05	—	—	—
Current history	4.56 ± 8.86	—	—	—
Total history	42.60 ± 51.32	—	—	—
Dysfunctional Attitudes	156.02 ± 28.36	124.03 ± 26.47	9.94	**<0.001**
A	6.27 ± 2.17	6.36 ± 1.26	-0.46	0.65
B	5.46 ± 2.07	6.15 ± 1.89	-2.97	**<0.001**
C	3.98 ± 1.88	5.70 ± 1.82	-7.90	**<0.001**
E	4.65 ± 1.78	5.06 ± 1.42	-2.23	**0.03**
F	4.67 ± 2.06	6.58 ± 2.08	-7.91	**<0.001**
G	4.33 ± 1.80	5.16 ± 1.71	-4.05	**<0.001**
H	5.00 ± 1.46	6.11 ± 1.41	-6.58	**<0.001**
I	6.27 ± 1.64	6.23 ± 1.57	0.21	0.84
L	5.38 ± 1.59	3.87 ± 1.73	7.85	**<0.001**
M	4.53 ± 1.54	4.60 ± 1.50	-0.35	0.73
N	5.95 ± 1.41	5.98 ± 1.29	-0.20	0.84
O	7.74 ± 1.71	5.23 ± 1.99	11.67	**<0.001**
Q1	4.97 ± 1.63	4.90 ± 1.67	0.37	0.71
Q2	5.46 ± 1.85	5.23 ± 1.51	1.22	0.22
Q3	4.63 ± 1.52	5.71 ± 1.32	-6.44	**<0.001**
Q4	7.14 ± 1.50	4.98 ± 1.85	10.87	**<0.001**

Bold values indicate statistical significance

*SD *Standard Deviation

### Correlations of anxiety facets of 16PF and dysfunctional attitudes and subtypes of MDD and HC groups

Pearson’s correlations demonstrated significant associations between specific domains of 16 PF and dysfunctional attitudes in both MDD group and HC group (see Tables 2 and 3). Cohen’s (1992) standards for Pearson’s correlation coefficient effect size were used to determine the strength of the effects (i.e., small, 0 ≤ *r* < .3; medium, 0.3 ≤ *r* < .5; large, 0.5 ≤ *r* ≤ 1) [5]. As expected, the 16PF dimensions related to the dysfunctional attitudes were mainly concentrated in the four anxiety facets of 16 PF. Within the MDD group, specifically, C factor showed a medium negative correlation with dysfunctional attitudes (*r* = − .35, *p* < .001), whereas other three primary personality traits were positively associated with dysfunctional attitudes with small to medium correlation: L factor (*r* = .16, *p* = .036), O factor (*r* = .40, *p* < .001), and Q4 factors (*r* = .30, *p* < .001).

**Table 2:** Pearson’s correlation coefficients of 16PF and dysfunctional attitudes and subtypes in the MDD group (*n* = 168)

	1	2	3	4	5	6	7	8	9	10	11	12	13	14	15	16	17	18	19	20	21	22	23	24	25
DAS Total score	-																								
Vulnerability	0.74^*^	-																							
Attraction and repulsion	0.77^*^	0.52^*^	-																						
Perfectionism	0.73^*^	0.55^*^	0.44^*^	-																					
Compulsion	0.62^*^	0.40^*^	0.40^*^	0.46^*^	-																				
Seeking applause	0.73^*^	0.41^*^	0.53^*^	0.47^*^	0.34^*^	-																			
Dependence	0.74^*^	0.48^*^	0.56^*^	0.50^*^	0.37^*^	0.50^*^	-																		
Self-determination attitude	0.74^*^	0.58^*^	0.49^*^	0.56^*^	0.41^*^	0.43^*^	0.47^*^	-																	
Cognition philosophy	0.33	0.12	0.19^*^	− 0.06	0.07	0.26^*^	0.17^*^	0.05	-																
A	**− 0.15** ^*^	**− 0.14** ^*^	− 0.04	− 0.17	− 0.05	− 0.13	− 0.01	**− 0.13** ^*^	− 0.12	-															
B	− 0.08	− 0.04	**− 0.14** ^*^	− 0.03	**− 0.23** ^*^	− 0.08	0.01	0.06	0.01	− 0.01	-														
C	**− 0.35** ^*^	**− 0.24** ^*^	**− 0.28** ^*^	**− 0.26** ^*^	− 0.07	**− 0.30** ^*^	**− 0.27** ^*^	**− 0.23** ^*^	**− 0.20** ^*^	0.34^*^	− 0.16^*^	-													
E	− 0.04	0.01	0.01	**− 0.15** ^*^	0.01	− 0.03	− 0.03	− 0.03	0.01	− 0.03	− 0.16^*^	0.26^*^	-												
F	− 0.09	0.02	− 0.02	− 0.11	0.02	− 0.07	− 0.02	**− 0.17** ^*^	− 0.09	0.22^*^	− 0.04	0.34^*^	0.52^*^	-											
G	**− 0.22** ^*^	**0.15** ^*^	**− 0.24** ^*^	**− 0.15** ^*^	− 0.17	**− 0.24** ^*^	− 0.06	− 0.10	**-0.14** ^*^	0.30^*^	− 0.01	0.28^*^	0.04	0.12	-										
H	**− 0.17** ^*^	0.08	− 0.06	− 0.12	**− 0.16** ^*^	− 0.11	**− 0.13** ^*^	**− 0.14** ^*^	**− 0.14** ^*^	0.12	− 0.08	0.38^*^	0.36^*^	0.45^*^	0.15	-									
I	0.07	0.11	0.01	0.02	− 0.01	0.09	0.03	0.03	0.12	− 0.01	0.26^*^	− 0.11	− 0.03	0.05	0.04	− 0.01	-								
L	**0.16** ^*^	**0.16** ^*^	0.11	0.07	0.06	**0.18** ^*^	**0.15** ^*^	0.04	0.12	0.03	− 0.03	− 0.17^*^	0.05	− 0.05	0.01	− 0.19^*^	0.03	-							
M	− 0.12	0.08	0.07	**− 0.19** ^*^	− 0.02	− 0.11	− 0.02	**− 0.17** ^*^	0.04	0.25^*^	− 0.02	0.17^*^	0.13	0.27^*^	− 0.01	0.08	0.12	− 0.17^*^	-						
N	− 0.04	− 0.04	− 0.10	− 0.03	− 0.07	− 0.04	0.03	− 0.02	0.04	− 0.07	0.01	− 0.03	0.15	0.10	0.02	0.04	− 0.03	− 0.06	− 0.01	-					
O	**0.40** ^*^	**0.21** ^*^	**0.28** ^*^	**0.23** ^*^	**0.17** ^*^	**0.31** ^*^	**0.30** ^*^	**0.29** ^*^	**0.35** ^*^	− 0.12	0.09	− 0.37^*^	− 0.06	− 0.30^*^	0.01	− 0.24^*^	0.21^*^	0.20^*^	− 0.24^*^	− 0.02	-				
Q1	− 0.08	− 0.10	0.03	− 0.06	− 0.08	-0.12	− 0.01	− 0.04	− 0.08	0.28^*^	− 0.01	0.08	0.01	0.03	0.06	0.07	− 0.10	0.00	0.19^*^	− 0.06	− 0.01	-			
Q2	− 0.03	0.01	0.01	0.01	− 0.07	− 0.04	**− 0.17** ^*^	0.04	0.01	− 0.24^*^	0.15	− 0.21^*^	− 0.28^*^	− 0.19^*^	− 0.04	− 0.15	0.10	− 0.03	0.01	− 0.07	− 0.08	− 0.01	-		
Q3	− 0.13	− 0.01	**− 0.17** ^*^	− 0.09	**− 0.15** ^*^	**− 0.14** ^*^	− 0.12	0.10	− 0.11	0.04	0.12	0.24^*^	0.04	0.03	0.25^*^	0.25^*^	− 0.02	− 0.03	− 0.05	− 0.01	− 0.19^*^	− 0.02	− 0.03	-	
Q4	**0.30** ^*^	**0.14** ^*^	**0.19** ^*^	**0.13** ^*^	**0.24** ^*^	**0.28** ^*^	**0.18** ^*^	**0.17** ^*^	**0.25** ^*^	− 0.34^*^	− 0.08	− 0.25^*^	0.03	− 0.21^*^	− 0.13	− 0.28^*^	0.11	0.20^*^	− 0.28^*^	0.04	0.49^*^	− 0.15	− 0.10	− 0.13	1

Bold values indicate statistical significance

*DAS *Dysfunctional Attitude Scale

^*^P < .1

**Table 3:** Pearson’s correlation coefficients of 16PF and dysfunctional attitudes and subtypes in the HC group (*n *= 130).

	1	2	3	4	5	6	7	8	9	10	11	12	13	14	15	16	17	18	19	20	21	22	23	24	25
DAS Total score	-																								
Vulnerability	0.67^*^	-																							
Attraction and repulsion	0.75^*^	0.56^*^	-																						
Perfectionism	0.79^*^	0.58^*^	0.56^*^	-																					
Compulsion	0.57^*^	0.29^*^	0.29^*^	0.37^*^	-																				
Seeking applause	0.69^*^	0.26^*^	0.36^*^	0.43^*^	0.33^*^	-																			
Dependence	0.81^*^	0.43^*^	0.58^*^	0.60^*^	0.48^*^	0.51^*^	-																		
Self-determination attitude	0.65^*^	0.39^*^	0.38^*^	0.57^*^	0.36^*^	0.34^*^	0.49^*^	-																	
Cognition philosophy	0.28^*^	0.06	0.23^*^	− 0.01	− 0.01	0.14	0.15	− 0.12	-																
A	− 0.02	− 0.07	0.04	0.03	0.01	**− 0.18** ^*^	0.01	0.12	− 0.02	-															
B	0.01	− 0.10	− 0.05	0.07	− 0.03	0.04	0.03	0.03	− 0.02	− 0.17^*^	-														
C	**− 0.34** ^*^	**− 0.20** ^*^	**− 0.36** ^*^	**− 0.22** ^*^	− 0.07	**− 0.23** ^*^	**− 0.25** ^*^	− 0.11	**− 0.28** ^*^	0.17	− 0.19^*^	-													
E	− 0.06	− 0.05	− 0.03	− 0.10	− 0.02	**− 0.17** ^*^	0.08	**0.15** ^*^	− 0.11	0.20^*^	− 0.11	0.18^*^	-												
F	**− 0.18** ^*^	− 0.11	**− 0.24** ^*^	− 0.09	− 0.12	− 0.06	− 0.14	0.08	**− 0.28** ^*^	0.19^*^	− 0.04	0.48^*^	0.31^*^	-											
G	− 0.10	− 0.12	**− 0.15** ^*^	0.00	0.04	− 0.09	− 0.01	0.09	**− 0.28** ^*^	0.11	− 0.03	0.38^*^	− 0.01	0.08	-										
H	**− 0.21** ^*^	− 0.14	**− 0.21** ^*^	− 0.14	− 0.09	− 0.14	**− 0.15** ^*^	0.01	**− 0.25** ^*^	0.18^*^	− 0.19^*^	0.43^*^	0.37^*^	0.55^*^	0.17	-									
I	0.05	− 0.11	− 0.07	0.01	0.12	0.10	0.05	0.03	0.08	− 0.02	0.15	− 0.18^*^	− 0.13	− 0.07	0.01	− 0.15	-								
L	**0.33** ^*^	**0.31** ^*^	**0.37** ^*^	**0.24** ^*^	0.10	**0.19** ^*^	**0.28** ^*^	0.09	**0.16** ^*^	− 0.05	0.10	− 0.30^*^	0.07	− 0.29^*^	− 0.03	− 0.20^*^	0.04	-							
M	− 0.04	− 0.09	− 0.07	− 0.06	0.01	− 0.01	− 0.01	0.12	− 0.13	0.16	− 0.14	0.21^*^	0.18^*^	0.15	− 0.03	0.19^*^	0.13	− 0.00	-						
N	0.08	− 0.07	**0.18** ^*^	0.12	− 0.03	0.01	0.12	− 0.01	0.06	0.06	0.03	0.20^*^	0.13	0.05	− 0.06	0.08	0.01	− 0.06	0.06	-					
O	**0.41** ^*^	**0.33** ^*^	**0.42** ^*^	**0.27** ^*^	0.10	**0.27** ^*^	**0.24** ^*^	0.13	**0.35** ^*^	− 0.11	0.07	− 0.63^*^	− 0.24^*^	− 0.48^*^	− 0.22^*^	− 0.48^*^	0.16	0.39^*^	− 0.19^*^	− 0.00	-				
Q1	− 0.03	− 0.05	0.03	− 0.03	− 0.05	− 0.04	− 0.05	− 0.02	0.06	0.16	− 0.23^**^	0.04	0.13	0.03	0.02	0.11	− 0.05	0.09	0.28^*^	− 0.02	− 0.06	-			
Q2	− 0.04	− 0.03	− 0.07	0.01	**− 0.18** ^*^	− 0.01	− 0.06	− 0.02	0.13	− 0.16	0.14	− 0.20^*^	− 0.07	− 0.29^*^	− 0.15	− 0.31^*^	0.16	0.21^*^	0.04	− 0.06	0.08	0.08	-		
Q3	**− 0.30** ^*^	**− 0.26** ^*^	**− 0.37** ^*^	− 0.12	**− 0.21** ^*^	− 0.09	**− 0.27** ^*^	− 0.08	**− 0.20** ^*^	0.01	0.04	0.42^*^	0.10	0.18^*^	0.32^*^	0.25^*^	− 0.18^*^	− 0.17^*^	− 0.01	0.02	− 0.32^*^	− 0.04	− 0.06	-	
Q4	**0.51** ^*^	**0.45** ^*^	**0.49** ^*^	**0.35** ^*^	**0.26** ^*^	**0.30** ^*^	**0.39** ^*^	**0.22** ^*^	**0.24** ^*^	− 0.12	0.08	− 0.60^*^	− 0.16	− 0.50^*^	-0.15	− 0.52^*^	0.05	0.39^*^	− 0.27^*^	− 0.16	0.67^*^	− 0.04	0.18^*^	− 0.32^*^	-

Bold values indicate statistical significance

*DAS *Dysfunctional Attitude Scale

^*^P<0.1

Within the HC group, C factor were negatively correlated with dysfunctional attitudes with a medium correlation (*r* = − .34, *p* < .001), the L factor (*r* = .33, *p* < .001) and O factor (*r* = .41, *p* < .001) were positively correlated with dysfunctional attitudes in a medium range, and Q4 factor (*r* = .51, *p* < .001) showed a strong positive correlation with dysfunctional attitudes.

### Hierarchical regression analyses of 16 PF on dysfunctional attitudes and subtypes of MDD and HC groups

Table 4 shows the results of the hierarchical regression analyses of 16 PF on dysfunctional attitudes and subtypes in the MDD group. The 16PF dimensions which predicted dysfunctional attitudes score were C factor (*β* = − 0.19), G factor (*β* = − 0.16), and O factor (*β* = 0.26), with a ΔR^2^ of 21%. Additionally, the predictive effect of certain 16PF dimension on certain DAS subtype was found. Specifically, G factor and O factor would predict attraction and repulsion, C factor would predict perfectionism, B factor would predict compulsion, C factor and G factor would predict seeking applause, C factor and O factor would predict dependence, and G factor and O factor would predict cognition philosophy.

**Table 4:** Hierarchical regression analyses of 16PF on dysfunctional attitudes and subtypes in the MDD group (*n* = 168)

Standard coefficient									
	DAS Total score	Vulnerability	Attraction and repulsion	Perfectionism	Compulsion	Seeking applause	Dependence	Self-determination attitude	Cognition philosophy
A	-0.01	-0.10	—	—	—	—	—	0.06	—
B	—	—	-0.11	—	**-0.20**	—	—	—	—
C	**-0.19**	-0.16	-0.16	**-0.18**	—	**-0.17**	**-0.21**	-0.10	0.01
E	—	—	—	-0.11	—	—	—	—	—
F	—	—	—	—	—	—	—	-0.03	—
G	**-0.16**	-0.09	**-0.20**	-0.06	—	**-0.17**	—	—	**-0.17**
H	-0.03	—	—	—	-0.15	—	-0.05	-0.04	-0.02
L	0.05	0.11	—	—	—	0.10	0.07	—	—
M	—	—	—	-0.06	—	—	—	-0.04	—
O	**0.26**	0.14	**0.23**	0.11	0.05	0.15	**0.19**	0.19	**0.32**
Q2	—	—	—	—	—	—	-0.16	—	—
Q3	—	—	-0.06	—	-0.10	0.01	—	—	—
Q4	0.05	-0.07	-0.01	-0.02	0.13	0.08	-0.02	-0.03	0.10
ΔR2	0.21	0.10	0.17	0.10	0.11	0.16	0.13	0.09	0.17

Bold values indicate statistical significance. ΔR2 indicates the changes R2 of the model from level 2 to level 3. The three hierarchies of the regression model were as follows: level 1: sex, age, education; level 2: duration of total episodes, duration of current episodes, episode counts, HAMA, HAMD_24_; level 3: 16PF factors that show a significant relationship with DAS total score and each subtype

The results of the hierarchical regression analysis of the HC group were displayed in Table 5 The 16PF dimensions which predicted dysfunctional attitudes were L factor (*β* = 0.19) and Q4 factor (*β* = 0.44), with a ΔR^2^ of 25%. The predictive effects of certain 16PF dimension on certain DAS subtype were as follows: C factor, L factor, and Q4 factor would predict vulnerability; L factor, N factor, Q3 factor, and Q4 factor would predict attraction and repulsion; Q4 factor would predict compulsion; Q2 factor and Q4 factor would predict compulsion; L factor and Q4 factor would predict dependence; E factor and Q4 factor would predict self-determination attitude; and O factor would predict cognition philosophy.

**Table 5:** Hierarchical regression analyses of 16 PF on dysfunctional attitudes and subtypes in the healthy control group (*n* = 130)

Standard coefficient									
	DAS Total score	Vulnerability	Attraction and repulsion	Perfectionism	Compulsion	Seeking applause	Dependence	Self-determination attitude	Cognition philosophy
A	—	—	—	—	—	-0.07	—	—	—
C	0.09	**0.25**	-0.06	-0.09	—	-0.05	-0.08	—	0.01
E	—	—	—	—	—	-0.11	—	**0.20**	—
F	0.10	—	0.05	—	—	—	—	—	-0.09
G	—	—	0.05	—	—	—	—	—	-0.17
H	0.13		0.12	—	—	—	0.13	—	-0.06
L	**0.19**	**0.19**	**0.21**	0.17	—	0.13	**0.19**	—	0.02
N	—	—	**0.26**	—	—	—	—	—	—
O	0.18	0.08	0.08	0.08	—	0.11	-0.02	—	**0.29**
Q2	—	—	—	—	**-0.25**	—	—	—	—
Q3	-0.09	-0.15	**-0.19**	—	-0.09	—	-0.13	—	-0.01
Q4	**0.44**	**0.44**	**0.41**	**0.29**	**0.26**	0.17	**0.39**	**0.23**	-0.15
ΔR2	0.25	0.22	0.35	0.13	0.11	0.10	0.15	0.07	0.13

Bold values indicate statistical significance. ΔR2 indicates the changes R2 of the model from level 2 to level 3. The three hierarchies of the regression model were as follows: level 1: sex, age, education; level 2: HAMA, HAMD_24_; level 3: 16PF factors that show a significant relationship with DAS total score and each subtype

## Discussion

From the authors’ knowledge, the present study is the first study to investigate the relationship between 16PF and dysfunctional attitudes. This study found that specific domains of 16PF were significantly associated with dysfunctional attitudes. As hypothesized, the anxiety facets of 16PF were especially associated with dysfunctional attitudes in both MDD and HC groups, with overall stronger correlation coefficients compared to other primary personality traits. It was consistent with the previous findings in Big Five (NEO PI-R) model that neuroticism, which clearly corresponded to the anxiety facets of 16PF [6], was strongly linked to the dysfunctional attitudes in depression [14, 15]. Additionally, the inter-group comparison suggested that a higher level of anxiety feature indicating a low score of C factor, a high score of L factor, a high score of O factor, and a high score of Q4 factor, have been found in the MDD group compared to the HC group. It has broadened the previous finding of the significant associations between neuroticism facets and the symptoms of depression [16].

However, regression analysis found that the predictive effect of the certain anxiety facet on dysfunctional attitudes differentiated between the MDD group and HC group. This study found C factor and O factor are the major personality dimensions associated with dysfunctional attitudes in the MDD group, and L factor and Q4 factor are secondary; while L factor and Q4 factor are the major personality dimensions associated with dysfunctional attitudes in the HC group, and C factor and O factor are secondary. One possible explanation for this finding is that dysfunctional attitudes, driven by low self-esteem and self-doubt, might be more likely to be associated with MDD, while dysfunctional attitudes driven by distrust of others and releasing drive/energy, might be less likely to linked to MDD. However, future research will allow verification of this explanation.

In addition, this finding added a new scope in the discrepancy between people with or without depression, indicating that the anxiety patterns lead to dysfunctional attitudes between depressed individuals and healthy people might be different. People in MDD group might show anxious patterns of unstable emotion, worries, poor self-worth, low self-esteem, self-doubting, and insecure feelings [10], which leads to their dysfunctional attitudes. It has been indicated that people who have emotional stability showing excellent ability of emotional control, and can remain calm and effectively manage their emotions under pressure [17]. Whereas neurotic people who have unstable emotion and worries showing low levels of self-esteem and confidence, which might lead to the dysfunctional attitudes, beliefs and behavior in their lives [18]. Whereas healthy people might show the anxiety patterns of a higher level of psychological discomfort, distrust of others, and emotional regulation difficulties under stressful situation, which leads to their dysfunctional attitudes.

These findings suggested that each of the four dimensions of the anxiety facet has its own unique significance, which is also the unique advantage of 16PF, allowing us to conduct a more comprehensive personality assessment.

## Limitations

The present study is strengthened by investigating the relationship between personality traits and dysfunctional attitudes based on a comprehensive framework of personality in the comparable samples of clinical and non-clinical participants. The present study located dysfunctional attitudes and subtypes within the sixteen in-depth personality traits, in order to better understand how dysfunctional attitudes might develop.

However, there are still some limitations in the present study. First, the current sample could only represent a limited range of adult population in China and the results should be considered with cautiousness. Thus, future studies should use a representative sample including children, adolescents, and elders from broader areas. Second, there are limited literatures of this field of research, therefore the results of this study need to be retested in the future in order to provide more reliable conclusions. Third, this study has not investigated if there is an interrelationship among the anxiety facets of 16PF, dysfunctional attitudes, and depression. A further research is needed to examine whether dysfunctional attitudes could be a potential process that mediates the effect of anxiety-related personality traits on depression [19]. Additionally, the in-depth examination of the effect of relevant specific domains of 16PF on dysfunctional attitudes should be further conducted and explain why the results differ between the MDD group and HC group.

## Conclusions

The present findings indicated that personality traits, especially the anxiety-related personality traits such as emotionally instability, distrust of others, worried status, distrust of others, and emotional regulation difficulties under stressful situations, might be the important variables to explain the development of dysfunctional attitudes in people with or without MDD. However, the anxiety facets of 16PF which could explain dysfunctional attitudes and the subtypes differentiates between the MDD group and HC group. It might suggest that the anxiety patterns of MDD patients and healthy individuals are different, and future studies are needed to explain the difference on the framework of personality traits.

## Data Availability

The datasets used and/or analysed during the current study available from the corresponding author on reasonable request.
